# Dispersive Liquid–Liquid Microextraction (DLLME) and LC-MS/MS Analysis for Multi-Mycotoxin in Rice Bran: Method Development, Optimization and Validation

**DOI:** 10.3390/toxins13040280

**Published:** 2021-04-15

**Authors:** Sofiyatul Akmal Salim, Rashidah Sukor, Mohd Nazri Ismail, Jinap Selamat

**Affiliations:** 1 Laboratory of Food Safety and Food Integrity, Institute of Tropical Agriculture and Food Security, Universiti Putra Malaysia, Serdang 43400, Selangor, Malaysia; jinap@upm.edu.my; 2 Analytical Biochemistry Research Centre, Universiti Sains Malaysia, Minden 11800, Pulau Pinang, Malaysia; mdnazri@usm.my; 3 Department of Food Science, Faculty of Food Science and Technology, Universiti Putra Malaysia, Serdang 43400, Selangor, Malaysia

**Keywords:** dispersive liquid–liquid microextraction, multi-mycotoxin, liquid chromatography mass spectrometry, response surface methodology, rice bran

## Abstract

Rice bran, a by-product of the rice milling process, has emerged as a functional food and being used in formulation of healthy food and drinks. However, rice bran is often contaminated with numerous mycotoxins. In this study, a method to simultaneous detection of aflatoxins (AFB1, AFB2, AFG1, and AFG2), ochratoxin A (OTA), deoxynivalenol (DON), fumonisins (FB1 and FB2), sterigmatocystin (STG), T-2 toxin, HT-2 toxin, diacetoxyscirpenol (DAS) and zearalenone (ZEA) in rice bran was developed, optimized and validated using dispersive liquid–liquid microextraction (DLLME) and liquid chromatography-tandem mass spectrometry (LC-MS/MS). In DLLME, using a solvent mixture of methanol/water (80:20, *v*/*v*) as the dispersive solvent and chloroform as the extraction solvent with the addition of 5% salt improved the extraction recoveries (63–120%). The developed method was further optimized using the response surface methodology (RSM) combined with Box–Behnken Design (BBD). Under the optimized experimental conditions, good linearity was obtained with a correlation coefficient (*r*^2^) ≥ 0.990 and a limit of detection (LOD) between 0.5 to 50 ng g^−1^. The recoveries ranged from 70.2% to 99.4% with an RSD below 1.28%. The proposed method was successfully applied to analyze multi-mycotoxin in 24 rice bran samples.

## 1. Introduction

In Asia, more than 600 million tonnes of rice and 124 million tonnes of rice by-products are produced annually [1]. Rice bran constitutes 10% of the rough rice weight and may vary depending on the pre-treatment type and the degree of milling in rice milling factory [2]. Rice bran is generally used as an ingredient in poultry feeds, as it contains 12–22% oil, 11–17% protein, 6–14% fibre, 10–15% moisture, and 8–17% ash [2]. Moreover, rice bran is also rich in vitamins, minerals and bioactive components, contributing to health benefits for humans, and is considered as a functional food [3,4]. Albeit the benefit, rice bran is often susceptible to mycotoxin contamination due to the growth of toxin-producing fungi at the aleurone layer, which may contain a large population of *Aspergillus flavus* [5]. *Aspergillus, Fusarium,* and *Penicillium* were among the listed fungal genera which are responsible for mycotoxin contamination in rice and rice by-products [6]. High incidence of *A. flavus* and *A. parasiticus* in rice by-products, including rice bran, was previously reported [7,8,9]. 

The occurrence of mycotoxins in food including rice bran is a serious health issue and should be controlled and minimized. Hence, it is crucial to have a rapid, sensitive, robust and cost-effective technique to detect the presence of mycotoxins in rice bran. Various methods have been employed for the determination of mycotoxins, including high-performance liquid chromatography (HPLC), gas chromatography-mass spectrometry (GC-MS), and enzyme-linked immunosorbent assay (ELISA) [10]. Additionally, liquid chromatography-tandem mass spectrometry (LC-MS/MS) has been widely used in the determination of mycotoxins in various complex matrices due to its high sensitivity and ability to detect multiple mycotoxins in a single analysis [11,12,13,14,15,16,17]. LC-MS/MS is the most promising instrumental method for multi-mycotoxin determination. It is highly selective and can perform screening, confirmation and quantitation of hundreds of analytes simultaneously. 

The sample pre-treatment procedure is crucial to improve the sensitivity and selectivity of the analytical method. In recent years, dispersive liquid-liquid microextraction (DLLME) has emerged as a contemporary technique in sample preparation. It is simple, effective and considered a green microextraction technique. This is because it employs a minimal volume of solvents and a mixture of organic solvents that act as extraction and dispersive solvents. These two solvents are rapidly injected into a sample (in an aqueous), which produces turbulence and subsequently forms fine droplets. The fine droplets (sedimented at the bottom of the tube) are then injected into a chromatographic system [18]. In mycotoxin analysis, DLLME was employed in numerous types of sample matrices such as determination of aflatoxins in herbal tea [19], mycotoxins in peanuts [20], multi-mycotoxin in fruit juices [21], fumonisins in maize [22] and mycotoxins in human urine [23]. In DLLME, several factors may affect the extraction efficiency, including the type of extraction and dispersive solvents, as well as the addition of salt for the salting-out process. Hence, optimization is vital to obtain the optimal condition to extract the target analytes from a sample. Response surface methodology (RSM) is a statistical technique that is used to optimize the performance or response of a process by determining design factors settings. It investigates the interaction effects between several factors at different levels. In this study, RSM was employed in the DLLME method optimization, as it offers several advantages, including a minimum number of experiments, saves time and cost, and minimal use of chemicals [24,25,26].

The aim of this study is to establish a simple method for multi-mycotoxin determination, namely AFB1, AFG1, AFB2, AFG2, STG, T-2, HT-2, ZEA, DAS, FB1, FB2, DON and OTA by employing the DLLME method to assess rice bran. Using Box–Behnken Design (BBD) with three factors and three levels, the experimental parameters affecting the extraction and DLLME efficiency were optimized with RSM. The optimized method was then validated and successfully applied to determine the occurrence and quantity of multi-mycotoxin in rice bran samples collected from rice milling industries and commercial sources.

## 2. Results and Discussion

### 2.1. Liquid Chromatography Mass Spectrometry (LC-MS/MS) Optimization

**Figure 1:**
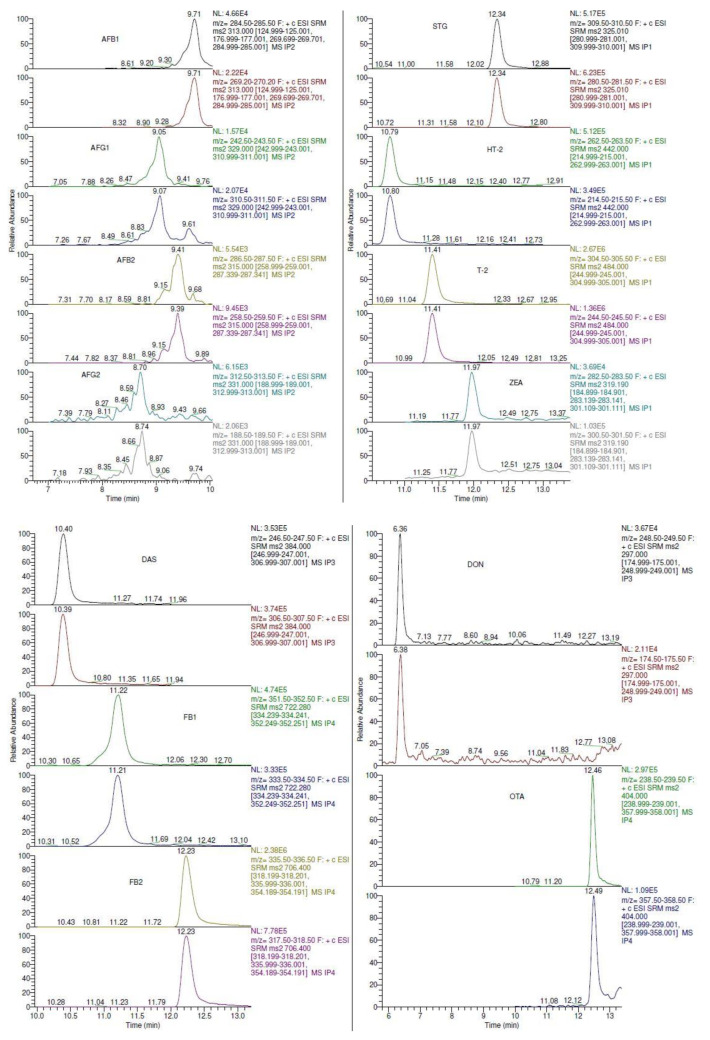
Total ion chromatogram of target analytes using selected reaction monitoring (SRM) as listed in Table 1.

The mass spectrometry (MS) method development was conducted via the introduction of target analytes into the mass spectrometry system by direct infusion of the standard solutions. All of the target analytes were found to be more sensitive in the positive mode. Previous studies have demonstrated that most mycotoxins are likely to produce [M+H]^+^ [16,27,28,29,30,31]. Full scan MS/MS was performed to examine the fragmentation of all target analytes. The most intense ion in the fragmentation was selected for quantitation and the second intense ion was chosen for qualification. According to the European Commission (2002), at least two ion transitions should be used for the monitoring of each target analyte in an instrumental method [32]. Most of the product ions used for the quantification of the mycotoxins had been previously reported in the literature [16,33,34,35]. Selected reaction monitoring (SRM) was established in a positive ion mode with optimized collision energy (CE) and tube lens values for each target analyte (Table 1). The chromatograms obtained using the SRM are shown in Figure 1. The mycotoxin analytes were eluted from 8.82 to 12.64 min, with AFG2 and STG being the first and last peaks, respectively. Twelve mycotoxins were separated within 18 min, including the re-equilibration step. High resolution, together with peak asymmetry and minimal baseline noise, demonstrates the satisfactory selectivity of the LC-MS/MS method.

In liquid chromatography, the selection of mobile phases is essential to achieve high sensitivity and good resolution. Different mobile phases, namely water (solvent A) and methanol (MeOH, solvent B) versus water (solvent A) and acetonitrile (MeCN, solvent B), were examined by injecting mixed standard solutions into the LC-MS/MS system. It was observed that all analytes exhibited acceptable results using MeOH as compared to MeCN, with no significant differences. These results were consistent with those of other studies [28,29,35,36,37,38,39], which suggested that MeOH was the optimal mobile phase in multi-mycotoxin analysis using LC-MS/MS system. Furthermore, MeOH is less expensive and more environmentally friendly in view of laboratory waste disposal, and hence have been preferred by many [40]. 

**Figure 2:**
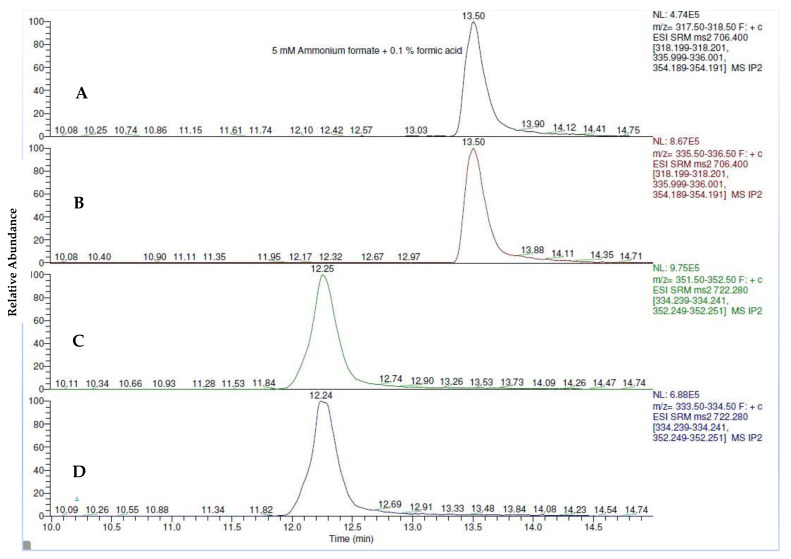
Chromatograms of fumonisin B2 (FB2) (**A**,**B**) and fumonisin B1 (FB1) (**C**,**D**) using 5 mM ammonium formate with 0.1% of formic acid as the mobile phase.

To achieve a good separation, different mobile phase additives (0.1% formic acid, 0.1% acetic acid, 5 mM ammonium formate and 5 mM ammonium acetate) were examined using Accucore C18 column. Ammonium formate (5 mM) with 0.1% formic acid (A) and methanol with 0.1% formic acid (B) were selected as mobile phases in this study. Sun et al. (2019) indicated that ammonium formate used as additives in mobile phases improved the ion signals of target analytes in LC-MS/MS [15]. As shown in the peak resolution of fumonisins, it was improved through the addition of 0.1% formic acid into ammonium formate as the mobile phase (Figure 2).

This result corroborates with the finding of Kiontke et al. (2016), who suggested that formic acid, as an ion-pairing reagent, helped to improve the sensitivity of target analytes, especially when the positive mode of electrospray ionization (ESI) was used [41]. Significant improvements in the peak asymmetry factor, a reduction of 15% in peak widths and an increment of 30% in peak capacity were obtained in LC-MS/MS analysis when ammonium formate and formic acid were used as mobile phase modifiers [42].

The optimization of LC-MS/MS was performed using Thermo Scientific Accela liquid chromatography system attached to a Thermo Scientific TSQ Quantum Access MAX mass spectrometer. Nevertheless, due to certain limitations, the reproducibility of the developed methods in other similar instruments were not tested. Thus, it is worth noting that further optimization should be conducted elsewhere to fit other LC-MS/MS applications. 

### 2.2. Dispersive Liquid–Liquid Microextraction (DLLME)

Due to complex food matrices, sample pre-treatment is usually required as a clean-up step prior to instrumental analysis. The purification or concentration, or both, of the sample, helps to improve the selectivity and sensitivity of the method. In this study, DLLME was used as a sample clean-up. To achieve the optimal DLLME conditions for determination of multi-mycotoxin in rice bran, several experimental parameters which affect the efficiency of DLLME (such as type and volume of extraction solvent, salt addition and volume of water) were investigated.

#### Selection of Dispersive Solvent, Extraction Solvent and Addition of Salt for Extraction of Mycotoxin in Rice Bran

**Figure 3:**
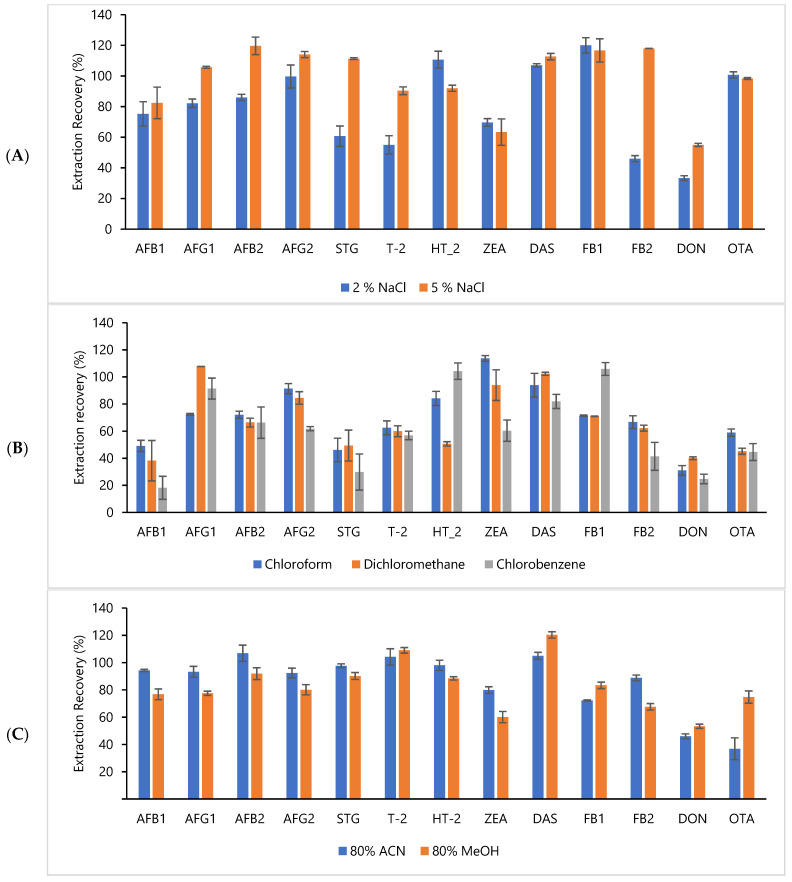
Recoveries of target analytes using different (**A**) dispersive solvent; 80% MeCN in water and 80% MeOH in water, (**B**) extraction solvents; chloroform, dichloromethane and chlorobenzene, and (**C**) salt concentrations; 2% and 5%. Data are expressed as mean ± SD of triplicates (n = 3).

In DLLME, the selection of the dispersive solvent is the most crucial part when it comes to ensuring the efficiency of the sample extraction. A dispersive solvent should be miscible with both the extraction solvent and the aqueous phase. In this study, 80% MeCN and 80% MeOH were tested and used as dispersive solvents. Triplicates of the spiked sample (AFB1 and AFG1, 2 ng mL^−1^; AFB2 and AFG2, 6 ng mL^−1^; OTA, 5 ng mL^−1^; FB1, FB2 and ZEA, 100 ng mL^−1^; DAS, HT-2, T-2 and STG, 10 ng mL^−1^) were analyzed using different dispersive solvents, and the recoveries of all target analytes were calculated and are illustrated in Figure 3A. OTA extracted with MeCN had the lowest recovery, of 37% while other target analytes’ recoveries ranged from 46 to 107%. On the other hand, the recoveries of target analytes using MeOH ranged from 60 to 120%. This finding was in agreement with that produced by Campone et al. (2014), who used a mixture of MeOH/H_2_O as a dispersive solvent [43].

The type of extraction solvent is the most crucial and essential step in the DLLME procedure. It has to fulfil several criteria, i.e., the high capability to extract the target analytes; less soluble in water; capable of solubilising in the dispersive solvent, and able to form an emulsion in the presence of the dispersive solvent [34]. During the preliminary experiment, several halogenated solvents, including chloroform (CHCl_3_), dichloromethane (CH_2_Cl_2_) and chlorobenzene (C_6_H_5_Cl), were tested. CHCl_3_ produced the highest recoveries, ranging from 46 to 114%, and it was selected as the extraction solvent in the DLLME analysis (Figure 3B). The findings of the present study were consistent with those of other researchers [43,44,45] who used CHCl_3_ as the extraction solvent. Due to the suitability and capability of CHCl_3_ to extract mycotoxin from food products such as fruit juices, beer, wine, honey, milk, maize, rice, wheat products, cereals and flour, it was widely used as the extraction solvent in the food matrices. CHCl_3_ has a high density in water, is able to form turbidity, has low solubility in water, and exhibits good chromatographic behaviour, while it is also able to extract mycotoxin from food matrices [18]. 

The salting-out effect on extraction efficiency was created by introducing 2% and 5% of NaCl in the DLLME analysis. The recoveries of target analytes using different percentages of salt are illustrated in Figure 3C. The result showed that the recoveries of T-2, STG, AFG1, AFG2, AFB1, AFB2 and FB2 increased when the percentage of salt in the water increased. Thus, the introduction of salt at 5% was applied in the DLLME procedure to improve the extraction efficiency. The present findings were consistent with those of other reports, which produced the highest mycotoxin recovery from the rice sample through the addition of NaCl into the water in DLLME [46].

This study did not report DON because after using MeOH as the dispersive solvent, CHCl_3_ as the extraction solvent and 5% of NaCl showed unsatisfactory low recovery ranging between 31 and 55%. In DLLME, the ionization form of analyte depends on the pH of the aqueous solution. This may have an impact on water solubility and extractability. The ionized form is soluble in water, whereas non-ionized easily transferred into DLLME extractant [47]. A previous study indicated that the most suitable pH value for DON was at 11 [48]. In this study, however, the pH was maintained at pH 3 and resulted in the unsatisfactory recovery of DON. Therefore, it was decided to exclude DON in optimization and validation.

### 2.3. Optimising of DLLME by Box-Behnken Approach and Response Optimization Using Composite Desirability

The multivariate statistical technique was employed using RSM to minimize the number of experiments, shorten the analysis time, and reduce the cost [49]. Preliminary experiments showed that all of the variables examined in this study affected the target analytes’ intensities (peak height). Therefore, the effects of three variables, including volume of extraction solvent, the concentration of salt, and volume of water, were examined using BBD. In this study, analysis of variance (ANOVA) was conducted, and the quadratic models for all target analytes (except for STG and OTA) were found to be significant at the 95% confidence level (*p* < 0.05). A low *p*-value indicates that the combined effects of all independent variables contributed significantly to maximising the response. Meanwhile, the lack-of-fit values were greater than 0.05 (between 0.137 and 0.907), thus suggesting that the models were adequate to predict the response variables. The values of the determination coefficient (*R*^2^) were between 0.775 and 0.957, which implied that the sample variation of 77.5 to 95.7% of intensities (peak height) was attributable to the independent variables. The quadratic equations and other parameters are shown in Table 2.

**Figure 4:**
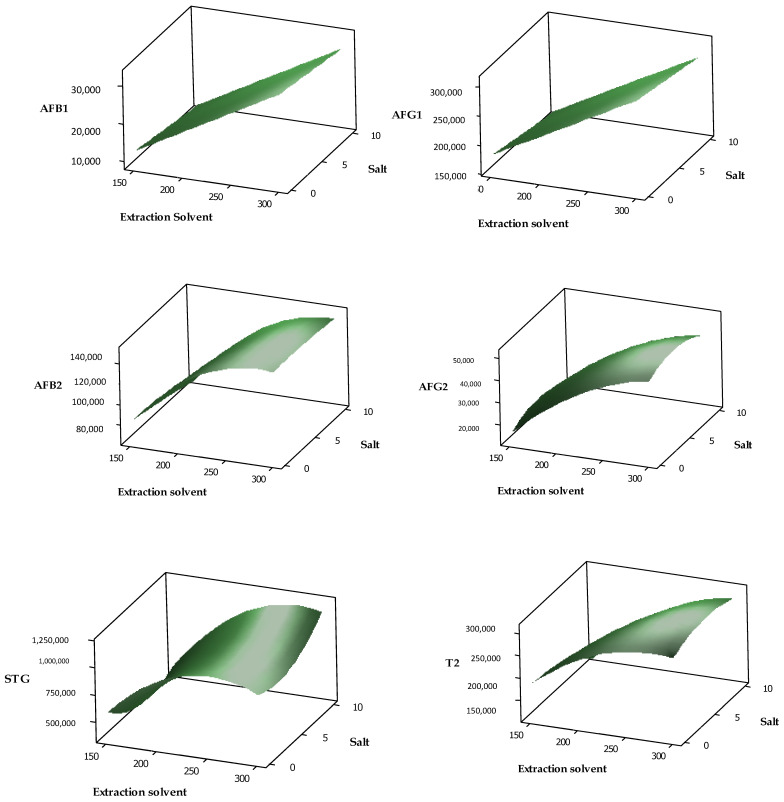
Surface plot of peak height versus extraction solvent volume and salt percentage for aflatoxins (AFB1, AFB2, AFG1, and AFG2), sterigmatocystin (STG) and T-2 toxin (T-2).

**Figure 5:**
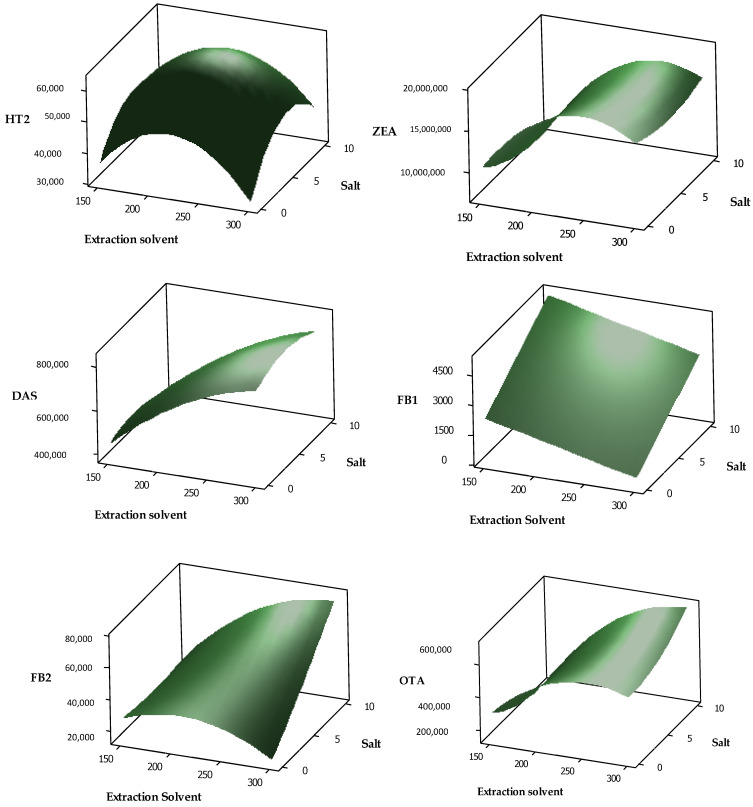
Surface plot of peak height versus extraction solvent volume and salt percentage for HT-2 toxin (HT-2), zearalenone (ZEA), diacetoxyscirpenol (DAS), fumonisins (FB1 and FB2), and ochratoxin A (OTA).

The effects of the independent variables and their interactions with the target analytes’ response could be graphically described using three-dimensional response surface plots. The surface plots in Figure 4 and Figure 5 show a relationship between the volume of extraction solvent and concentration of salt, which affected the peak height in the chromatogram (intensity) of each target analyte. The peak height of AFB1, AFG1, AFB2, AFG2, T-2, DAS, STG, FB2 and OTA increased as the volume of extraction solvent and percentage of salt increased. This was in accordance with published reports, where the volume of extraction solvent affected the target analytes’ responses significantly [19,34,43]. The composite desirability was employed in this analysis to obtain the optimal values that could be achieved for all evaluated variables. Based on the desirability results, the predicted optimal DLLME conditions were the volume of extraction solvent (chloroform), 295 µL; concentration of salt, 10%; and volume of water, 3 mL. Triplicates of blank samples spiked with target analytes were analyzed using the predicted optimal conditions during verification. The result indicated that good recoveries and low RSD values were obtained for all target analytes. 

**Figure 6:**
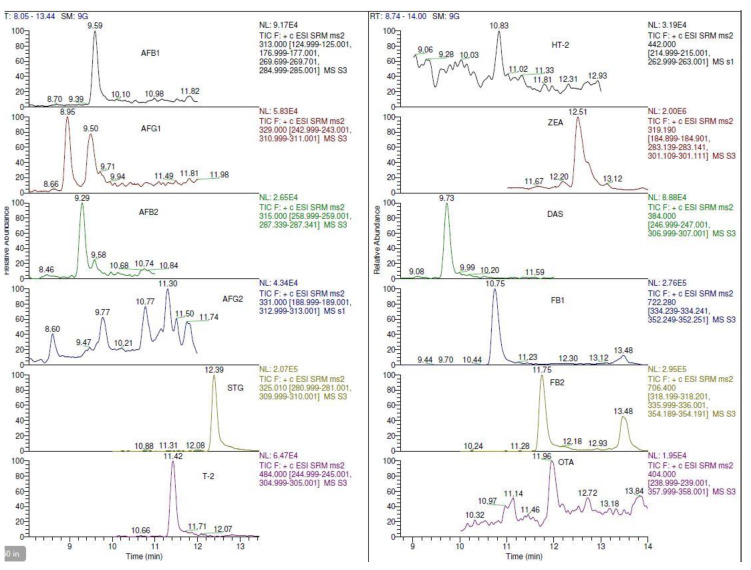
Total ion chromatogram of target analytes after optimization at concentration of 2 ng g^−1^ for AFB1 and AFG1, 6 ng g^−1^ for AFB2 and AFG2, 5 ng g^−1^ for OTA, 100 ng g^−1^ for FB1, FB2 and 10 ng g^−1^ for ZEA DAS, HT-2, T-2 and STG.

Table 3 summarises the peak height (relative abundance) for each target analyte before and after optimization. The table shows that the response surface model had a significant effect on the peak height of the target analytes and can be adapted in DLLME analysis for multi-mycotoxin determination. Figure 6 shows the total ion chromatogram of target analytes after optimization.

### 2.4. Validation

#### 2.4.1. Specificity and Matrix-Effects (ME)

**Figure 7:**
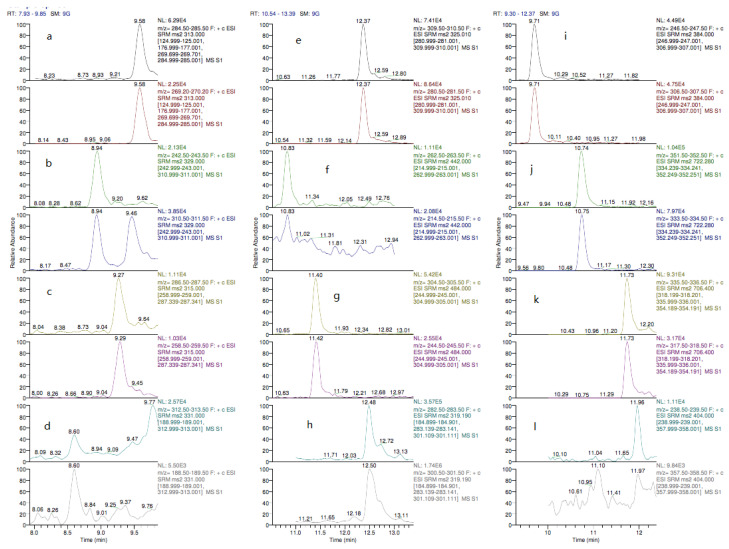
Chromatogram of a spiked sample with multi-mycotoxin at limit of quantitation (LOQ) levels; (**a**) AFB1 (1.5 ng g^−1^), (**b**) AFG1 (1.5 ng g^−1^), (**c**) AFB2 (3.0 ng g^−1^), (**d**) AFG2 (3.0 ng g^−1^), (**e**) STG (5.0 ng g^−1^), (**f**) T-2 (5.0 ng g^−1^), (**g**) HT-2 (5.0 ng g^−1^), (**h**) ZEA (5.0 ng g^−1^), (**i**) DAS (5.0 ng g^−1^), (**j**) FB1 (150 ng g^−1^) (**k**) FB2 (75 ng g^−1^) and (**l**) OTA (3.75 ng g^−1^).

In accordance with EU Regulation 2002/657/EC [32], the method was developed using two SRM transition and exclusively identified by retention time or SRM transition, or both. Figure 7 shows the chromatogram of the blank sample spiked with multi-mycotoxin. Multi-mycotoxin were well separated between 8.6 to 12.5 minutes. AFG2 was eluted first and ZEA was the last before started with the equilibrium of the column. The retention time depends on the molecular structure, the nature of mobile and stationary phases, the flow rate and column dimensions [50]. There was an absence of interferences in the monitored chromatogram of multi-mycotoxin confirming the high selectivity of the method [51]. This shows that the developed method was able to distinguish between the multi-mycotoxin and the interference based on signals generated under actual experimental conditions [52]. 

**Figure 8:**
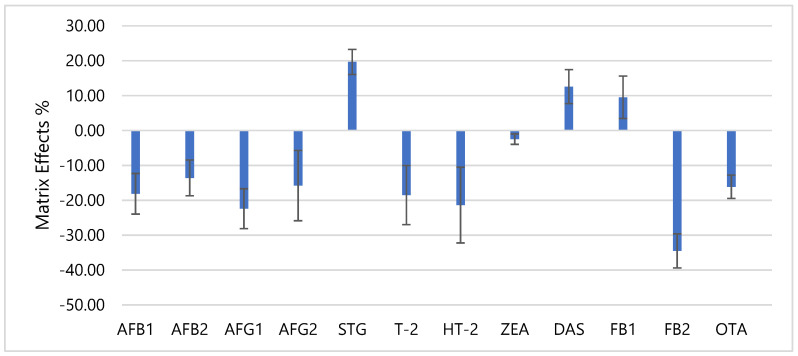
Matrix effects of each mycotoxin at 1.5 ng g^−1^ for AFB1 and AFG1, 3.0 ng g^−1^ for AFB2 and AFG2, 5.0 ng g^−1^ for STG, T-2, HT-2, ZEA and DAS, 150 ng g^−1^ for FB1, 75 ng g^−1^ for and 3.75 ng g^−1^ for OTA.

Matrix-effects (ME) was evaluated by comparing the peak responses of the standard mycotoxins (n = 5) spiked in the extraction solvent with the spiked rice bran samples at LOQ levels for each analyte. The ME on different analytes is shown (Figure 8). Generally, ME% ranging from −20% to + 20% indicated that the signal enhancement or suppression is acceptable [19,52,53]. It can be seen that, for AFB1, B2, G2, STG, T2, ZEA, DAS, FB1 and OTA (−2.47 to 19.7%) were within the acceptable range. However, AFG1, HT-2 and FB2 (−21.4 to −30%) were slightly out of the acceptable range. This indicated that the signals of the analytes were suppressed by 21.4 to 30% in rice bran matrix. Nevertheless, to compensate these significant ME and to improve the linearity, reliability and accuracy of the analytical results matrix-matched calibration curves were used [54].

#### 2.4.2. Limit of Detection (LOD), Limit of Quantitation (LOQ) and Linearity

The LOD, LOQ and linearity of the mycotoxins are listed in Table 4. The ranges for linearity used in this study were narrow due to some limitations for the mycotoxin standards. However, this is not unusual as a linear range for LC-MS instruments is normally fairly narrow, as shown in [31,55]. A linear range can be expanded in a variety of ways, including using an isotope-labeled internal standard, lowering the flow rate in the ESI source to reduce charge competition, or using a nano-ESI. Nevertheless, these options were not explored in the study. The limit of detection (LOD) and LOQ of mycotoxins in rice bran were in the range of 0.5 to 150 ng g^−1^, which rectified the sensitive level to meet the requirement of the European Union (EU) regulations for the corresponding maximum levels of mycotoxins in cereals [56]. The coefficient correlation (*R*^2^) greater than 0.99 was achieved for all mycotoxins. Coefficient correlation of more than 0.9 indicates a very strong relationship between two observed variables [57]. 

#### 2.4.3. Accuracy, Precision and Recovery

Accuracy and precision were evaluated by analysing blank samples spiked at the LOQ level as listed in Table 5. Seven replicates of these samples and six points’ matrix-matched calibration samples were analyzed by a single analyst in three different days. Recovery and standard deviation (SD) were calculated for each of the mycotoxins. 

#### 2.4.4. Occurrence of Multi-Mycotoxin in Industrial and Commercial Samples

Twenty-four rice bran samples (n = 24) were subjected to the developed, optimized and validated method for determination multi-mycotoxin using DLLME and LC-MS/MS. Nineteen samples (n = 19) were collected from rice milling factories in the state of Penang, Perak and Kedah, Malaysia while five samples were purchased commercially. The occurrence of multi-mycotoxin was calculated and presented in Table 6. The result showed that 42% of rice bran samples were positive with at least a single mycotoxin at concentration between 1.69 to 157.44 ng g^−1^. All the positive samples were obtained from rice industries, while no mycotoxins were found in commercial samples. Twenty-one percent (21%) of the samples were contaminated with AFG2 with the highest concentration at 8.07 ng g^−1^, followed by AFB1 (17%) at 2.19 ng g^−1^ as the maximum concentration. Eight percent (8%) of the rice bran samples were found to contain AFB2. According to European Commission (2006), the maximum level for AFB1 in cereals and products derived from cereals is 2 ng g^−1^ while the maximum level for sum of AFB1, AFB2, AFG1 and AFG2 was set at 4 ng g^−1^. About 90% of the positive samples were contaminated with aflatoxins above the maximum level. Four percent (4%) of samples were contaminated with FB1 and FB2. However, the sum of the mycotoxins in the positive samples was below the maximum level, i.e., 200 ng g^−1^.

## 3. Conclusions 

This paper describes a simple and fast method with which to detect multi-mycotoxin in rice bran using DLLME as the sample extraction, the selection of different disperser solvent, extraction solvent and the effect of salt addition prior to analysis by LC-MS/MS. Direct infusion of a standard mixture was followed by a full scan of MS/MS and SRM in a positive mode for each target analyte, in combination with formic acid in ammonium formate, and methanol was shown to be encouraging in terms of acceptable and satisfactory performances for chromatographic separations. RSM with BBD employed was shown to be an effective and simplified approach for cost and time saving, and to examine the optimal combination of tested variables. With a nominal volume of extraction solvent, mid concentration of salt and minimal volume of water were suggested by the composite desirability; these results showed a successful applicability in extracting multi-mycotoxin from rice bran with good range of recoveries. Under the optimal conditions, the developed method presented satisfactory validation characteristics (linearity, recovery and precision) and successfully applied for the determination of multi-mycotoxin in rice bran samples. 

## 4. Materials and Methods 

### 4.1. Chemicals and Reagents

Chemicals and reagents used in this study were of HPLC and analytical grade. HPLC-grade methanol (MeOH), acetonitrile (MeCN), chloroform (CHCl_3_), dichloromethane (CH_2_Cl_2_) and sodium chloride were purchased from Fisher Scientific (Loughborough, UK). HPLC-grade chlorobenzene (C_6_H_5_Cl), analytical-grade formic acid (FA) and ammonium formate (NH_4_HCO_2_) were obtained from Acros Organic (Fair Lawn, NJ, USA). Aflatoxin mixture (B1 and G1, 1 µg mL^−1^; B2 and G2, 0.3 µg mL^−1^), STG (50 µg mL^−1^), T-2 toxin (10 µg mL^−1^), HT-2 toxin (10 µg mL^−1^), ZEA (10 µg mL^−1^) and DAS (10 µg mL^−1^) standards were obtained from Sigma Aldrich (St. Louis, MO, USA). FB1 (50 µg mL^−1^), FB2 (56 µg mL^−1^), DON (10 µg mL^−1^) and OTA (9 µg mL^−1^) were purchased from Fermentek (Jerusalem, Israel). 

Stock of mixed standards were divided into three; mixed standard A was prepared by diluting AFs mixture, mixed standard B was prepared by diluting of FB1, FB2, OTA, DON and STG stock standard and mixed standard C was prepared by diluting trichothecene mixed solution. The standards were diluted using HPLC-grade methanol. The final concentration of mixed standard A for AFB1 and AFG1 were 50 ng mL^−1^; AFB2 and G2 were 15 ng mL^−1^; mixed standard B for FB1, FB2, and STG and were 10 µg mL^−1^ and OTA was 1 µg mL^−1^ and mix standard C was 1 µg mL^−1^. All the standards were stored at −20 °C prior to analysis.

Spiked sample was prepared by spiking mix mycotoxin standards A, B and C into rice bran sample. The final concentrations of the spiked sample were AFB1 and AFG1 (2 ng g^−1^), AFB2 and AFG2 (6 ng g^−1^), OTA (5 ng g^−1^), FB1, FB2, DON and ZEA (100 ng g^−1^), DAS, HT-2, T-2 and STG (10 ng g^−1^).

Commercial rice bran, purchased from the local market in Penang, Malaysia was used as a blank sample in method development and optimization. The sample was absent in multi-mycotoxin during the screening analysis in the preliminary study. For safety reason, all the glassware used were soaked overnight in 10% sodium hypochlorite. 

### 4.2. Instrumental and Analytical Conditions

Sample analysis was performed on Accela™ ultra-high-performance liquid chromatography (UHPLC) system (Thermo Scientific, Waltham, MA, USA) with an automatic sample injector and quaternary pump. The HPLC system was attached with TSQ Quantum Access MAX mass spectrometer equipped with an electrospray (HESI II) MAX-source. An Accucore C18 (column 2.1 mm ID × 100 mm L, 2.6 μm) (Thermo Scientific, Waltham, MA, USA) was used for chromatographic separations. The mass spectrometry was operated in positive mode and the ESI spray voltage was set to 3000 kV. The capillary temperature was 350 °C, sheath gas flow rate was set at 30 arbitrary units, aux valve flow 7.0 arbitrary units and ion sweep gas pressure 2.0 arbitrary units. 

LC-MS/MS method development was initiated by determining the ion of target analytes in a mass spectrometer. This was done by directly infused standard solution into the ion source using a syringe infusion pump. During this step, the MS parameters such as collision energy and tube lens value for each target analytes were observed to obtain the optimum condition for ionization and finally improved the sensitivity of the instrument. Full scan and full scan MS/MS were performed to examine the sensitivity and fragmentation of the target analytes. The most abundant product ion was selected for quantitation and the second abundant product ion for qualification. The selected reaction monitoring (SRM) method was developed in positive mode using 0.7 full-width half max (FWHM) isolation width together with optimized collision energies (CE) for each target analyte.

In liquid chromatography, the selection of mobile phase is important to achieve proper retention, peak shape of LC-MS/MS response and chromatographic separation [58]. In this study, MeOH and MeCN were tested, and sensitivity (response) and chromatographic separation were monitored. Further optimization was done by introducing different mobile phase additives; 0.1% formic acid, 0.1% acetic acid, 5 mM ammonium formate and 5 mM ammonium acetate in mobile phases. The retention factor (*k*) of each analyte was determined using the equation below:

(1) $$k={\frac{\left(t_{R}-t_{0}\right)}{t_{0}}}_{}$$

where *t_R_* is retention time and *t*_0_ is non-retention time.

In this study, 5 mM ammonium formate with 0.1% formic acid (A) and methanol with 0.1% formic acid (B) were finally selected as mobile phases. LC was operated using a gradient elution program. It was started with 5% B and increased to 95% (10 min) and maintained at isocratic elution (10–12 min). Finally, solution B was decreased to 5% and maintained until end of the run (10–18 min) to achieve equilibrium. The injection volume was set to 20 µL at 200 μL min^−1^ flow rate.

### 4.3. Sample Preparation

#### 4.3.1. Extraction Procedure

Determination of mycotoxin in rice bran samples was adopted from previous study with slight modification [18]. Twenty grams of rice bran samples were extracted with 80 mL of 80% MeOH and sonicated for 15 minutes. The pH value of the sample was adjusted to 3.0 to 3.2 using 5 mM HCl solution and centrifuged at 3000 rpm for 5 min and then proceeded to DLLME analysis.

#### 4.3.2. Dispersive Liquid–Liquid Microextraction Procedure

One mL of the 80% methanolic extract (disperser solvent) was aliquoted in a microcentrifuge tube and added with 200 µL CHCl_3_ (extraction solvent). The mixture was vortexed for 30 secs and then rapidly injected into a new tube containing 5 mL of water using Hamilton syringe. A cloudy solution consisted of water, MeOH and CHCl_3_ was formed. The solution was further centrifuge at 3000 rpm for 5 minutes. The sedimented layer (bottom layer) was transferred to the new tube using a pipette. The sample was evaporated to dryness under nitrogen a stream at room temperature. The sample was then reconstituted with 100 µL of 0.1% FA in 5 mM ammonium formate: 0.1% FA in H_2_O (1:1, *v*/*v*) and subjected to LC-MS/MS analysis. The method was evaluated and optimized by comparing different types of extraction solvent, dispersive solvent and salt addition in water to increase the efficiency of the extraction for all target analytes.

#### 4.3.3. Selection of Dispersive Solvent, Extraction Solvent and Addition of Salt

During the selection of dispersive solvent, two sets of samples consist of blank and spiked samples (triplicates) containing target analytes were analyzed using two different dispersive solvents; 80% MeOH and 80% MeCN. The spiked samples were used, i.e., one spiked before the extraction procedure and the other were spiked after the extraction procedure. Recoveries of the extraction using different dispersive solvents were compared. Selection of extraction solvent was conducted by analyzing three sets of samples consist of blank and spiked samples (triplicates). The analysis was started with 80% MeOH as the dispersive solvent and proceed with DLLME. In DLLME, different extraction solvents, i.e., chloroform (CHCl_3_), dichloromethane (CH_2_Cl_2_) and chlorobenzene (C_6_H_5_Cl) were tested and recoveries of the extractions were compared. To observe the salting-out effect in the samples, the introduction of salt at different percentages, i.e., 2% and 5 % in DLLME procedure were done using three sets of samples consisted of blank and spiked samples (n = 3). Recoveries of the extraction were calculated and compared.

#### 4.3.4. Response Surface Methodology Using Box–Behnken Design

A preliminary experiment was implemented to choose the types of extraction and dispersive solvent. Response surface methodology (RSM) was engaged to optimize the key parameters of DLLME which included (A) volume of extraction solvent, (B) concentration of salt and (C) volume of water, as shown in Table 7. Box-Behnken Design (BBD) was employed to optimize the combination of the three variables; consist of three factors and three levels. The matrix design includes 15 experiment runs performed in random order to evaluate the main effects of the factors. In the study, the peak heights of target analytes were used as responses. All analyses were observed using Minitab Statistical Software (Minitab^®^ 17.1.0, State College, PA, USA).

#### 4.3.5. Response Optimization

Response optimization is an efficient way to help identify the optimal condition from a combination of evaluated variables. It can determine the combination of input variable settings that optimize a set of responses by calculating an optimal solution and drawing an optimization plot. In this study, simultaneous optimization of multiple responses was carried out using Minitab Software to collect composite desirability (D). From D value, optimal settings in a set of responses were classified and ranged from zero to one. One illustrates the ideal case, while zero represents that one or more responses are outside of the acceptable limits. The composite desirability (D) was expressed as follow:

D = (*d*_1_*x d*_2_*x...x d_n_*) *^n^* = (∏ (*d_i_^wi^*)) ^w^ (2)

where *d_i_* was individual desirability for the *i*^th^ response from *i*_1_^st^ to *i*_15_^th^; *wi* importance of the *i*^th^ response; W was the sum of *w^i^* and *n* was the number of responses.

### 4.4. Validation

Method validation was performed according to the guidelines for the validation and verification of quantitative and qualitative test methods by the National of Testing Authorities Australia (2018), 2002/657/EC by the European Communities, and EUR 24105 and EC No. 401/2006 by the European Union. Parameters assessed in method validation were selectivity, limit of detection (LOD), limit of quantitation (LOQ), linearity, recovery and precision. The selectivity of the method was examined by subjecting the blank sample and spiked sample containing all mycotoxins at the LOQ levels into LC-MS/MS system. The presence of interferences, i.e., absence of any additional peaks at similar retention time as multi-mycotoxin) was observed from the result [59]. The developed method must free from interferences which can lead to false positive results [60]. Matrix-effects (ME) was evaluated by comparing the peak responses of the standard mycotoxins (n = 5) spiked in the extraction solvent with the spiked rice bran samples at LOQ levels for each analyte. The ME was calculated using the formula:

ME = (A2 − A1/A1) × 100(3)

where, A1 is the average peak height of the mycotoxins and A2 is the average peak height of mycotoxins spiked in blank rice bran. A comparison of an increasing or decreasing of the detector response can be observed from the positive or negative ME values. 

Limit of detection (LOD) was determined by using ten replicates of blank samples spiked with multi-mycotoxin at pre-determined LOD concentration and the mean response of the spiked samples was calculated. Signal-to-noise ratio (S/N) approach is usually being used in chromatographic methods. In this study, S/N was determined by comparison of the signal between known low concentration and blank sample with calculated acceptable reliability. The lowest concentration level at S/N = 3 after blank correction with the acceptable result was considered as LOD. For linearity, six points of calibration standards were prepared for each analytes in the concentration ranges of 0.5 to 150 ng g^−1^ and analyzed in three consecutive days. The coefficient correlation (*R*^2^) was obtained using the last square approach. Accuracy and precision were done to determine the repeatability or reproducibility of result to examine if they are close to each other in a series of measurements [60]. It was expressed by calculating the relative standard deviation (RSD) of replicate results. To study the method repeatability, the evaluation was done by analysing seven replicates of blank sample spiked with multi-mycotoxin at the LOQ levels. These samples were analyzed in three consecutive days. Recovery analysis was done to examine the amount (in percentage) of multi-mycotoxin recovered after the analytical procedure [61]. To study the method recovery, the evaluation was done by analysing blank matrix together with spiking known concentrations of multi-mycotoxin into the test matrix. The difference between both results were calculated.

## Figures and Tables

**Table 1:** Collision energy (CE) and tube lens values of the monitored target analytes used in the selected reaction monitoring (SRM) method.

Analyte	Polarity	Precursor Ion (*m*/*z*)	Product Ion (*m*/*z*)	Collision Energy (CE)	Tube Lens	Retention Time (min)
AFB1	+	313	**285**	21	94	9.7
			269	25		
AFG1	+	329	**243**	22	86	9.0
			311	25		
AFB2	+	315	**287**	25	95	9.4
			259	28		
AFG2	+	331	**313**	23	86	8.7
			189	35		
STG	+	325	**310**	25	76	12.3
			281	34		
HT-2	+	442	**263**	20	70	10.7
			215	20		
T-2	+	484	**305**	20	70	11.4
			245	20		
ZEA	+	319	**283**	8	86	11.9
			301	10		
DAS	+	384	**247**	20	70	10.4
			307	20		
FB1	+	722	**352**	34	126	11.2
			334	37		
FB2	+	706	**336**	34	120	12.2
			318	35		
DON	+	297	**249**	9	90	6.3
			175	23		
OTA	+	404	**239**	25	90	12.4
			358	20		

Quantifying ions indicated in bold.

**Table 2:** The quadratic equations used to optimize the key variable in dispersive liquid–liquid microextraction (DLLME) procedure.

Target Analyte	Final Equation	*R* ^2^	ANOVA *p*-Value	
			Model	Lack of Fit
AFB1	−718,670 + 7156 A − 15,649 B + 24,688 C − 11.86 A^2^+ 697 B^2^ − 1369 C^2^ + 20.6 AB − 85.2 AC + 191 BC	0.934	0.01	0.57
AFG1	44,372 + 1536 A − 1881 B − 17,849 C − 2.41 A^2^ −188 B^2^ + 402 C^2^ + 12.5 AB + 53.7 AC − 221 BC	0.946	0.01	0.43
AFB2	−59,869 + 1822 A + 120 B − 15,664 C − 3.79 A^2^ − 39 B^2^ + 126 C^2^ + 9.1 AB + 51.7 AC − 423 BC	0.938	0.01	0.21
AFG2	−4299 + 513 A + 2432 B − 11,286 C − 1.016 A^2^ − 215 B^2^ + 359 C^2^ − 1.0 AB + 24.7 AC− 61 BC	0.866	0.03	0.41
STG	−995,532 + 22,381 A − 144,405 B − 141,900 C − 44.8 A^2^ + 5697 B^2^ + 2733 C^2^ + 232 AB + 26 AC + 6119 BC	0.775	0.06	0.76
T-2	−112,185 + 3512 A − 16,011 B − 15,247 C − 6.71 A^2^ − 722 B^2^ − 274 C^2^ + 52.5 AB + 30.8 AC + 1289 BC	0.954	0.00	0.30
HT-2	58,093 + 768 A + 6157 B − 25,799 C − 2.66 A^2^ − 455 B^2^ + 728 C^2^ + 5.6 AB + 61.3 AC − 354 BC	0.865	0.04	0.91
ZEA	−28,806,469 + 407,356 A − 882,897 B − 475,031 C − 829 A^2^ + 43,282 B^2^ − 24,239 C^2^ + 2063 AB + 782 AC −27,500 BC	0.854	0.02	0.31
DAS	−43,534 + 6319 A + 32,240 B − 72,094 C − 10.44 A^2^ − 2154 B^2^ + 3002 C^2^ + 24 AB + 111 AC − 2973 BC	0.883	0.01	0.82
FB1	−54,518 + 408 A + 12,533 B + 9554 C − 1.046 A^2^ + 266.0 B^2^ − 801 C^2^ − 25.53 AB + 9.50 AC − 1002 BC	0.957	0.01	0.23
FB2	−724,670 + 9510 A − 151,680 B + 67,179 C − 25.6 A^2^ + 1067 B^2^ 12,128 C^2^ + 439 AB + 209 AC + 11,944 BC + 209 AC + 11,944 BC	0.829	0.04	0.75
OTA	−700,860 + 12,471 A − 140,637 B − 22,763 C − 24.5 A^2^ + 2232 B^2^ − 4309 C^2^ + 213 AB + 7 AC + 7 AC + 11,293 BC	0.807	0.05	0.81

A, volume of extraction solvent; B, concentration of salt; C, volume of water.

**Table 3:** Difference of peak height for target analytes before and after optimization.

Target Analyte	Peak Height	
	Before Optimization	After Optimization
AFB1	11,282 ± 1484	23,011 ± 1411
AFG1	12,186 ± 477	21,256 ± 1636
AFB2	5833 ± 928	10,256 ± 289
AFG2	3245 ± 725	10,460 ± 122
STG	12,847 ± 588	87,240 ± 1939
T-2	16,959 ± 3429	54,895 ± 1414
HT-2	19,540 ± 896	11,042 ± 1416
ZEA	47,244 ± 2849	136,034 ± 13,576
DAS	6958 ± 7393	43,421 ± 3427
FB1	3245 ± 382	76,253 ± 7302
FB2	12,847 ± 790	37,199 ± 7099
OTA	16,959 ± 101	10,154 ± 1529

Data are expressed as mean ± SD of duplicates (n = 2).

**Table 4:** Limit of detection (LOD), LOQ and linearity of mycotoxins.

Mycotoxin	LOD (ng g^−1^)	LOQ (ng g^−1^)	Linearity Range (ng g^−1^)	*R* ^2^
AFB1	0.5	1.5	0.5–1.5	0.992
AFG1	0.5	1.5	0.5–1.5	0.992
AFB2	1.0	3.0	1.5–4.5	0.992
AFG2	1.0	3.0	1.5–4.5	0.994
STG	2.5	5.0	2.5–7.5	0.990
T-2	2.5	5.0	2.5–7.5	0.990
HT-2	2.5	5.0	2.5–7.5	0.992
ZEA	2.5	5.0	2.5–7.5	0.992
DAS	2.5	5.0	2.5–7.5	0.993
FB1	50	150	50–150	0.990
FB2	25	75	25–75	0.991
OTA	1.25	3.75	2.5–7.5	0.993

**Table 5:** Accuracy, repeatability and reproducibility of mycotoxins.

Mycotoxin	Concentration (ng g^−1^)	Within Assay Precision (%) (n = 7)	Between Assay Precision (%) (n = 21)	Accuracy (%) (n = 21)	Recovery (%) ±SD (n = 7)
AFB1	1.5	11.6	11.3	113.7 (11.7)	74.6 ± 0.1
AFG1	1.5	11.1	9.1	115.1 (8.8)	80.5 ± 0.0
AFB2	3.0	15.3	15.1	112.5 (15.1)	70.2 ± 0.1
AFG2	3.0	16.2	16.1	112.8 (16.5)	74.8 ± 0.2
STG	5.0	17.9	18.5	74.6 (13.5)	99.4 ± 0.1
T-2	5.0	13.1	7.9	119.1 (9.8)	76.6 ± 0.2
HT-2	5.0	14.3	14.5	108.6 (13.8)	72.8 ± 0.9
ZEA	5.0	13.9	12.8	102.1 (1.1)	95.1 ± 0.9
DAS	5.0	14.2	7.9	62.3 (4.9)	90.1 ± 0.3
FB1	150	5.6	12.0	101.0 (8.1)	71.9 ± 0.8
FB2	75	6.5	14.6	73.1 (7.1)	82.5 ± 1.3
OTA	3.75	14.6	14.5	60.2 (14.6)	87.0 ± 0.2

Data are expressed as mean ± SD. Percent of coefficient of variation (CV) is calculated as standard deviation/mean *100, expressed as parentheses for accuracy.

**Table 6:** Occurrence of multi-mycotoxin in industrial samples.

Mean Concentration (ng g^−1^) ± SD (n = 2)						
	AFB1	AFG1	AFB2	AFG2	FB1	FB2
S2	1.69 ± 0.17	0.11 ± 0.01	0.33 ± 0.07	6.49 ± 1.38	157.44 ± 0.52	28.3 ± 0.91
S3	2.13 ± 0.50	0.10 ± 0.02	0.33 ± 0.01	2.70 ± 0.27	n.d.	n.d.
S5	1.08 ± 0.22	0.08 ± 0.01	0.37 ± 0.17	6.19 ± 0.91	n.d.	n.d.
S6	1.09 ± 0.29	0.12 ± 0.03	0.36 ± 0.04	8.07 ± 0.06	n.d.	n.d.
S7	1.67 ± 0.02	0.08 ± 0.07	0.36 ± 0.07	2.34 ± 0.59	n.d.	n.d.
S9	2.19 ± 0.21	0.07 ± 0.02	0.20 ± 0.05	1.45 ± 0.41	n.d.	n.d.
S17	0.34 ± 0.02	0.10 ± 0.01	2.72 ± 0.71	2.85 ± 0.27	77.70 ± 0.62	75.56 ± 0.73
S20	0.27 ± 0.04	0.12 ± 0.01	3.88 ± 1.60	5.41 ± 1.31	n.d.	n.d.
S21	0.34 ± 0.04	0.12 ± 0.05	1.74 ± 0.22	2.76 ± 0.30	n.d.	n.d.
S22	0.31 ± 0.07	0.14 ± 0.07	1.55 ± 0.96	6.39 ± 1.50	n.d.	n.d.

n.d. = not detected; less than limit of detection.

**Table 7:** Experimental ranges and levels of independent variables.

Variables	Symbol	Range and Level		
		Low	Central	High
Volume of extraction solvent (µL)	A	150	225	300
Concentration of salt (%)	B	0	5	10
Volume of water (mL)	C	3	6.5	10

## Data Availability

All datasets generated for this study are included in the article.
